# Feasibility of Submandibular Gland Preservation in cT1-2N0 Squamous Cell Carcinoma in the Floor of the Mouth

**DOI:** 10.3389/fonc.2020.00579

**Published:** 2020-04-21

**Authors:** Wei Du, Qigen Fang, Shanting Liu, Defeng Chen, Ruihua Luo, Xu Zhang

**Affiliations:** Department of Head Neck and Thyroid, Affiliated Cancer Hospital of Zhengzhou University, Henan Cancer Hospital, Zhengzhou, China

**Keywords:** oral squamous cell carcinoma, submandibular gland, survival analysis, level 1b, cervical lymph node metastasis

## Abstract

**Purpose:** Our goal was to analyze the feasibility of submandibular gland (SMG) preservation in cT1-2N0 floor of the mouth (FOM) squamous cell carcinoma (SCC) patients.

**Methods:** Patients with cT1-2N0 FOM SCC were retrospectively enrolled and divided into two groups according to the management of the SMG. Level 1b tissues were divided into six groups according to their location with respect to the SMG. The Kaplan-Meier method was used to calculate the locoregional control (LRC) and disease-specific survival (DSS) rates. A Cox model was used to determine the independent risk factors.

**Results:** Twenty-nine patients underwent SMG-preserving neck dissection, and lymph node metastasis occurred in the superior group in 3 of the 37 dissections with a prevalence of 8.1% and in the anterior group in 2 of the 37 dissections with a prevalence of 5.4%. In patients without SMG preservation, lymph node metastasis occurred in the superior group in 7 of the 137 dissections with a prevalence of 5.1% and in the anterior group in 6 of the 137 dissections with a prevalence of 4.4%. The only pattern of SMG involvement was invasion by positive lymph nodes. The 5-year LRC rates for patients with SMG preservation and patients with SMG excision were 84 and 73%, respectively, and the difference was not significant (*p* = 0.239). The 5-year DSS rates for patients with SMG preservation and patients with SMG excision were 88 and 84%, respectively, and the difference was not significant (*p* = 0.524).

**Conclusions:** In early-stage FOM SCC patients, SMG involvement is rare, the most common metastatic site in level 1b is the superior group, and SMG preservation does not decrease the LRC or DSS rates. Therefore, the findings suggest that there might be high feasibility of SMG-preserving neck dissection in cT1-2N0 FOM SCC.

## Introduction

Cervical lymph node metastasis is the most important prognostic factor in oral squamous cell carcinoma (SCC), and neck dissection is required in nearly all patients. Since the first introduction of neck dissection (1), excision of the submandibular gland (SMG) has been a necessary component of lymphadenectomy owing to the presence of afferent lymph nodes and its proximity to the primary lesion (2). With the advancement of anatomic research and functional surgery concepts, the SMG was found to be responsible for 70 to 90% of unstimulated saliva production, and subjective complaints associated with SMG resection have been described (3); then, a number of researchers suggested that there were no lymph nodes within the SMG and that metastasis in the SMG from oral SCC was extremely rare (4–7). Furthermore, numerous cancer centers performed SMG-preserving neck dissection, and conflicting results were reported. Cakir Cetin et al. (8), Razfar et al. (9), Subramaniam et al. (10), Agarwal et al. (11), and Ebrahim et al. (12) suggested the feasibility of SMG preservation in the early stage of oral SCC. Chen et al. (13) supported the viewpoint that SMG preservation could be performed in tongue/buccal SCC but not SCC in the floor of the mouth (FOM). Similarly, Lanzer et al. (14) described that patients with oral SCC could benefit from SMG preservation but that this was not true for patients with tongue or FOM SCC. These findings reflect the disagreement regarding whether SMG preservation is feasible in FOM SCC. On the other hand, radiotherapy is usually suggested for advanced-stage patients, and permanent salivary dysfunction can occur with as little as 35 Gy of radiation treatment. Therefore, the main goal of the current study was to analyze the feasibility of SMG preservation in cT1-2N0 FOM SCC patients.

## Patients and Methods

The Zhengzhou University institutional research committee approved our study, and all participants provided written informed consent for medical research prior to the initial treatment. All experiments were performed in accordance with the relevant guidelines and regulations.

The medical records of patients with surgically treated cT1-2N0 FOM SCC between January 2012 and December 2018 were retrospectively reviewed. The inclusion criteria included the following: the disease must be primary; the disease was re-staged as cT1-2N0M0 according to the 8th AJCC classification followed by palpation and ultrasound, CT, and MRI examinations; and information regarding follow-up could be obtained. Data regarding age, sex, TNM stage, operation records, perineural invasion (PNI), lymphovascular invasion (LVI), and follow-up were extracted and analyzed.

All pathologic sections were re-reviewed by at least two pathologists, and PNI was considered to be present if tumor cells were identified within the perineural space and/or nerve bundle; LVI was positive if tumor cells were noted within the lymphovascular channels (15–18). A cT1-2 tumor referred to a tumor whose long diameter was not more than 2 cm or ranged from 2 to 4 cm, and a cN0 neck referred to a neck that did not have any suspicious lymph nodes based on palpation and preoperative ultrasound, CT and MRI examinations. The indication for postoperative radiotherapy included neck lymph node metastasis and the presence of PNI or LVI.

In our cancer center, we began to attempt SMG presentation starting in 2012 in selected patients. Elective neck dissection (END) (levels I-III) was routinely performed in FOM SCC patients. The decision of whether to preserve the SMG was mainly based on the tumor location and size, the intraoperative findings regarding gross cancer infiltration of the SMG and the surgeon's preference. Level 1b fibrofatty tissues were divided into six groups according to their location with respect to the SMG. The superior group consisted of lymphous and adipose tissue located superior to the SMG. The inferior group consisted of lymphous and adipose tissue located inferior to the SMG. The anterior group consisted of lymphous and adipose tissue located anterior to the SMG. The posterior group consisted of lymphous and adipose tissue located posterior to the SMG. The superficial group consisted of lymphous and adipose tissue located superficial to the SMG. The deep group consisted of lymphous and adipose tissue located deep to the SMG. Tissues from the six groups were separately sent for pathologic analysis. Primary tumor excision with at least 1 cm margins was usually performed without lip splitting. After treatment, the patients were examined every 3 months during the first year, every 6 months during the second year, and once per year after the second year (15–18). Once disease recurrence was suspected, aspiration biopsy or incisional biopsy combined with other examinations was performed.

The Chi-square test and Student's *t*-test were used to compare the demographic and pathologic variables. The Kaplan-Meier method was used to analyze the locoregional control (LRC) rate and disease-specific survival (DSS) rate. The survival time was calculated from the date of surgery to the last follow-up or to the date of first locoregional recurrence or cancer-related death. All statistical analyses were performed using SPSS 20.0, and *p* < 0.05 was considered to be significant.

## Results

A total of 141 (105 male and 36 female) patients were enrolled for analysis, and the mean age was 62.2 (range: 41–78) years. There were 82 (58.2%) and 59 (41.8%) smokers and drinker, respectively. Bilateral neck dissection was performed in 33 (23.4%) patients. T1 and T2 clinical tumor stages were defined in 70 (49.6%) and 71 (50.4%) patients, respectively. Occult lymph node metastasis occurred in 22 (15.6%) patients, of whom 3 (13.6%, 3/22) patients had bilateral cervical metastasis. PNI was present in 20 (14.2%) patients, and LVI was present in 15 (10.6%) patients. Good, moderate, and poor tumor differentiation was present in 49 (34.8%), 73 (51.8%), and 19 (13.5%) patients, respectively. Negative margins were achieved in all patients. A total of 20 (14.2%) patients underwent postoperative radiotherapy.

There were 29 patients who underwent SMG-preserving neck dissection, of whom 8 patients underwent bilateral neck dissections, and 5 patients received postoperative radiotherapy. As described in Table 1, compared to patients with SMG excision, patients with SMG preservation were younger and had smaller tumors (both *p* < 0.05). There was no significant difference regarding the other variables between the two groups (all *p* > 0.05).

**Table 1:** Demographic and pathologic information in patients with or without submandibular gland (SMG) preservation.

**Variables**	**SMG preservation (*n* = 29)**	**Control group (*n* = 112)**	***p***
Age	52.4 (41–65)	64.8 (50–78)	<0.001
**Sex**			
Male	21 (72.4%)	84 (75.0%)	
Female	8 (27.6%)	28 (25.0%)	0.776
Smoker	16 (55.2%)	66 (58.9%)	0.715
Drinker	12 (41.4%)	47 (42.0%)	0.955
**Neck dissection**			
Unilateral	21 (72.4%)	87 (77.7%)	
Bilateral	8 (27.6%)	25 (22.3%)	0.551
**cT**			
T1	22 (75.9%)	48 (42.9%)	
T2	7 (24.1%)	64 (57.1%)	0.002
**pT**			
T1	21 (72.4%)	45 (40.2%)	
T2	8 (27.6%)	67 (59.8%)	0.002
**pN**			
N0	25 (86.2%)	94 (83.9%)	
N+	4 (13.8%)	18 (16.1%)	0.789
PNI	5 (17.2%)	15 (13.4%)	0.766
LVI	4 (13.8%)	11 (9.8%)	0.736
**Tumor differentiation**			
Well	10 (34.4%)	39 (34.8%)	
Moderate	14 (48.3%)	59 (52.7%)	
Poor	5 (17.2%)	14 (12.5%)	0.804
Radiotherapy	5 (17.2%)	15 (13.4%)	0.766

*Gray indicates the variable is significant*.

As described in Table 2, there were 37 dissections in 29 patients with SMG preservation, and 2 patients had bilateral level 1b metastasis. Lymph node metastasis occurred in the superior group in 3 of the 37 dissections with a prevalence of 8.1%, in the anterior group in 2 of the 37 dissections with a prevalence of 5.4%, and in the posterior group in 1 of the 37 dissections with a prevalence of 2.7%; there were no positive lymph nodes in the superficial or deep groups. There were 137 dissections in 112 patients with SMG excision, and 1 patient had bilateral level 1b metastasis. Lymph node metastasis occurred in the superior group in 7 of the 137 dissections with a prevalence of 5.1%, in the anterior group in 6 of the 137 dissections with a prevalence of 4.4%, in the posterior group in 4 of the 137 dissections with a prevalence of 2.9%, in the inferior group in 1 of the 137 dissections with a prevalence of 0.7%, and in the superficial group in 1 of the 137 dissections with a prevalence of 0.7%. There were no positive lymph nodes in the deep group of SMG tissues. Therefore, the overall superior, inferior, anterior, posterior, and superficial metastasis rates were 5.7, 0.6, 4.6, 2.9, and 0.6%, respectively.

**Table 2:** Metastasis pattern of level 1b lymph nodes in patients with or without submandibular gland (SMG) preservation.

**Sub-groups**	**SMG preservation group (*n* = 29, 37 dissections)**	**SMG excision group (*n* = 112, 137 dissections)**	**Total**
Superior	3 (8.1%)	7 (5.1%)	5.7%
Inferior	0	1 (0.7%)	0.6%
Anterior	2 (5.4%)	6 (4.4%)	4.6%
Posterior	1 (2.7%)	4 (2.9%)	2.9%
Superficial	0	1 (0.7%)	0.6%
Deep	0	0	0
SMG	–	0	0

In patients with SMG excision, SMG involvement was observed in 2 patients with a prevalence of 1.8%. The only invasion pattern was direct invasion by positive lymph nodes. There were no SMGs involved in invasion of the primary tumor.

**Figure 1:**
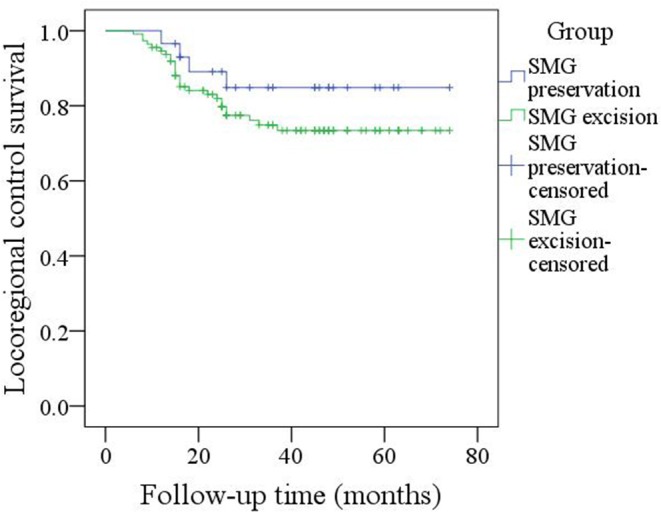
Comparison of locoregional control between patients with submandibular gland (SMG) preservation and patients with SMG excision (*p* = 0.239).

**Figure 2:**
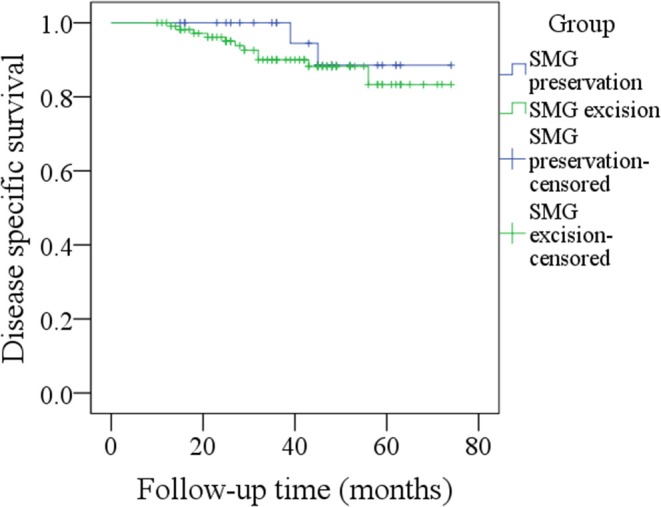
Comparison of disease-specific survival between patients with submandibular gland (SMG) preservation and patients with SMG excision (*p* = 0.524).

During our follow-up with a mean time of 35.7 months, there were 4 cases of locoregional recurrence and 2 cases of death in patients with SMG preservation as well as 26 cases of locoregional recurrence and 11 cases of death in patients with SMG excision. The 5-year LRC rates for patients with SMG preservation and patients with SMG excision were 84 and 73%, respectively, and the difference was not significant (*p* = 0.239, Figure 1). The 5-year DSS rates for patients with SMG preservation and patients with SMG excision were 88 and 84%, respectively, and the difference was not significant (*p* = 0.524, Figure 2). In further Cox model analysis Tables 3, 4, the factor of SMG preservation was not included because it was not significant in univariate analysis. Cervical lymph node metastasis (*p* < 0.001) and PNI (*p* = 0.008) were independent risk factors for LRC, and cervical lymph node metastasis (*p* < 0.001) and LVI (*p* = 0.005) as well as tumor differentiation were independent risk factors for DSS.

**Table 3:** Prognostic factors for the locoregional control in patients with cT1-2N0 squamous cell carcinoma of the floor of mouth.

**Variables**	**Univariate analysis**	**Cox model**	
	***p***	***p***	**RR[95%CI]**
Age (<62 vs. ≥62)	0.524		
Sex	0.356		
Smoking	0.287		
Drinking	0.296		
SMG preservation	0.239		
pT stage (T1 vs. T2)	0.005	0.184	3.664[0.786–12.004]
Neck node stage (N0 vs. N+)	0.001	<0.001	4.222[1.782–9.664]
Perineural invasion	0.014	0.008	2.847[1.471–7.552]
Lymphovascular invasion	0.008	0.085	3.412[0.925–9.227]
Tumor differentiation	0.152		
Well			
Moderate			
Poor			
Adjuvant treatment	0.411		

*Gray indicates the variable is significant*.

**Table 4:** Prognostic factors for the disease specific survival in patients with cT1-2N0 squamous cell carcinoma of the floor of mouth.

**Variables**	**Univariate analysis**	**Cox model**	
	***p***	***p***	**RR [95%CI]**
Age (<62 vs. ≥62)	0.285		
Sex	0.654		
Smoking	0.325		
Drinking	0.452		
SMG preservation	0.524		
pT stage (T1 vs. T2)	0.078		
Neck node stage (N0 vs. N+)	0.002	<0.001	2.222 [1.258–5.331]
Perineural invasion	0.236		
Lymphovascular invasion	0.014	0.005	2.338 [1.726–5.434]
Tumor differentiation	0.021		
Well			
Moderate		0.015	2.114 [1.235–4.002]
Poor		<0.001	4.669 [1.978–9.224]
Adjuvant treatment	0.444		

*Gray indicates the variable is significant*.

**Figure 3:**
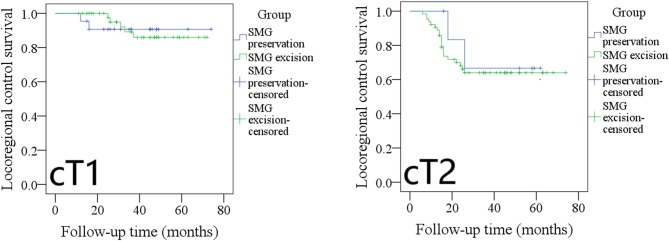
Comparison of locoregional control between cT1/cT2 patients with submandibular gland (SMG) preservation and patients with SMG excision (cT1: *p* = 0.953; cT2: *p* = 0.631).

During our subgroup analysis, the 5-year LRC rates in cT1 patients with SMG preservation and with SMG excision were 90 and 86%, respectively, and the difference was not significant (Figure 3, *p* = 0.953). The 5-year LRC rates in cT2 patients with SMG preservation and with SMG excision were 68 and 63%, respectively, and the difference was not significant (Figure 3, *p* = 0.631).

**Figure 4:**
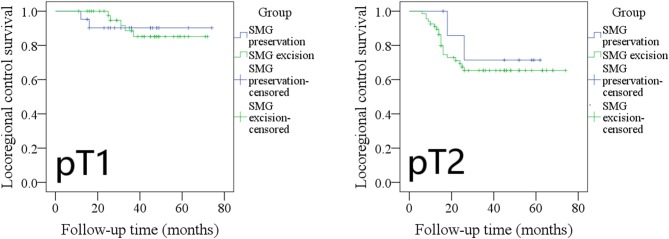
Comparison of locoregional control between pT1/pT2 patients with submandibular gland (SMG) preservation and patients with SMG excision (pT1: *p* = 0.961; pT2: *p* = 0.536).

The 5-year LRC rates in pT1 patients with SMG preservation and with SMG excision were 90 and 85%, respectively, and the difference was not significant (Figure 4, *p* = 0.961). The 5-year LRC rates in pT2 patients with SMG preservation and with SMG excision were 72 and 65%, respectively, and the difference was not significant (Figure 4, *p* = 0.536).

**Figure 5:**
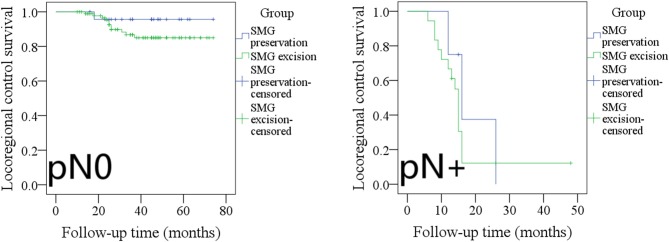
Comparison of locoregional control between pN0/pN+ patients with submandibular gland (SMG) preservation and patients with SMG excision (pN0: *p* = 0.243; pN+: *p* = 0.397).

The 5-year LRC rates in pN0 patients with SMG preservation and with SMG excision were 96 and 85%, respectively, and the difference was not significant (Figure 5, *p* = 0.243). The 2-year LRC rates in pN+ patients with SMG preservation and with SMG excision were 0 and 14%, respectively, and the difference was not significant (Figure 5, *p* = 0.397).

**Figure 6:**
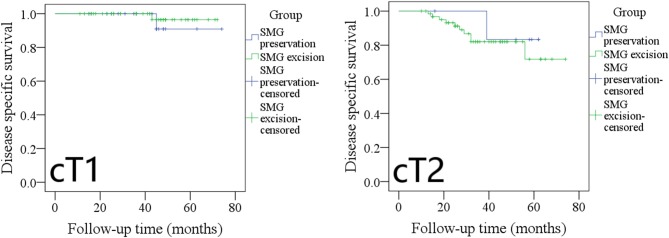
Comparison of disease-specific survival between cT1/cT2 patients with submandibular gland (SMG) preservation and patients with SMG excision (cT1: *p* = 0.534; cT2: *p* = 0.611).

The 5-year DSS rates in cT1 patients with SMG preservation and with SMG excision were 90 and 96%, respectively, and the difference was not significant (Figure 6, *p* = 0.534). The 5-year DSS rates in cT2 patients with SMG preservation and with SMG excision were 83 and 75%, respectively, and the difference was not significant (Figure 6, *p* = 0.611).

**Figure 7:**
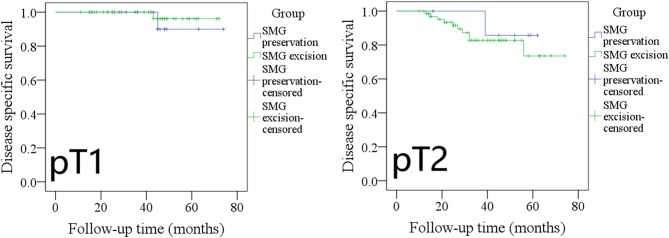
Comparison of disease-specific survival between pT1/pT2 patients with submandibular gland (SMG) preservation and patients with SMG excision (pT1: *p* = 0.527; pT2: *p* = 0.569).

The 5-year DSS rates in pT1 patients with SMG preservation and with SMG excision were 90 and 96%, respectively, and the difference was not significant (Figure 7, *p* = 0.527). The 5-year DSS rates in pT2 patients with SMG preservation and with SMG excision were 85 and 76%, respectively, and the difference was not significant (Figure 7, *p* = 0.569).

**Figure 8:**
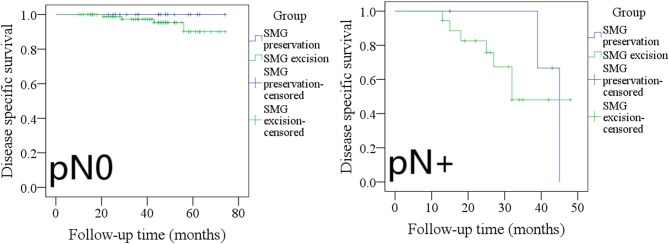
Comparison of disease-specific survival between pN0/pN+ patients with submandibular gland (SMG) preservation and patients with SMG excision (pN0: *p* = 0.275; pN+: *p* = 0.588).

The 5-year DSS rates in pN0 patients with SMG preservation and with SMG excision were 100 and 91%, respectively, and the difference was not significant (Figure 8, *p* = 0.275). The 3-year DSS rates in pN+ patients with SMG preservation and with SMG excision were 20 and 45%, respectively, and the difference was not significant (Figure 8, *p* = 0.588).

## Discussion

The most significant finding in the current study was that SMG preservation did not have an apparent negative effect on LRC or DSS in patients with cT1-2N0 FOM SCC; the only pattern of SMG involvement was the direct invasion of positive lymph nodes; the most common metastasis subgroup in level 1b metastasis was the superior group followed by the anterior group, and there were no metastases in the deep group of SMG tissues. Therefore, our study supports the high feasibility of SMG preservation in cT1-2N0 FOM SCC patients.

Rouviere et al. (19) described level 1b metastasis could be divided into five subgroups: the preglandular lymph nodes, the prevascular lymph nodes, the retrovascular lymph nodes, the retroglandular lymph nodes, and the intracapsular submandibular lymph nodes. DiNardo et al. (4) recently added another group of deep submandibular lymph nodes. There is great controversy regarding the existence of intracapsular and deep submandibular lymph nodes. Yang et al. (20) reviewed nearly 5,000 slides at 0.5 mm intervals from 116 oral SCC specimens and found that there were no deep or intrasubmandibular lymph nodes. Dhiwakar et al. (6) prospectively detected that neither the deep surgical bed nor the SMG after SMG excision contained any lymph nodes. Similar findings were also supported by Cakir Cetin et al. (8), Razfar et al. (9), and Chen et al. (21) as well as our group. Under these circumstances, it is possible for experienced surgeons to preserve the SMG during neck dissection.

Level 1b metastasis is the most common metastatic site in oral SCC (15, 22, 23), but only a few authors have analyzed the metastatic pattern in level 1b metastasis. DiNardo et al. (4) might have been the first to describe that 10 (24.4%) of 41 patients with FOM SCC had positive perivascular (superior) lymph nodes, and 4 patients had preglandular lymph node metastasis with a prevalence of 9.8%. This metastasis rate was significantly higher than our rate, and the most likely explanation is that only cT1-2 disease was analyzed in the current study. However, both studies indicated that the superior metastasis group represented the most common type in level 1b metastasis. Lim et al. (24) noted that in 14 patients with FOM SCC, 2 patients with T4 disease had occult superior metastasis, and no patients with early-stage disease had pathologic superior metastasis. The finding conflicted slightly with our finding. This variation might be due to the different sample sizes and racial differences. Spiegel et al. (25) analyzed the pattern of lymphatic metastasis in oral SCC and found that 7 of the 85 patients with cN0 disease had level 1b metastasis, and the most commonly involved site was the superior group followed by the anterior group, but there were no patients with FOM SCC included in the study. However, a similar phenomenon was also noted in the current study. This finding may provide benefits for the guidance of metastatic high-risk areas during SMG-preserving neck dissection.

There are three main models of SMG involvement based on current evidence. The first and most frequent is direct invasion by the tumor, which might account for 66–100% of cases (2), and FOM SCC has the highest probability of direct SMG invasion; however, in our study, no such phenomenon was observed. The accepted reason for this variation is that only cT1-2 disease was included in the analysis. The second model suggests SMG involvement through positive lymph nodes. Our finding was consistent with this mechanism, but the prevalence was as low as 1.8%. A review of previous similar studies showed that only 8 (0.6%) of 1,342 oral SCC patients demonstrated SMG invasion by metastatic level 1b lymph nodes (2, 9, 21, 25, 26). The third model suggests metastasis by intraglandular lymph nodes. The current study failed to note such a mechanism, and a similar finding was also reported by previous studies (9, 25, 26). In fact, we agree with the finding that metastatic disease in the SMG is more likely to be involved in cancer of non-head and neck origin (2). Recently, Fives et al. (5) introduced a fourth mechanism of tumor discovery, which was extremely rare, and no other similar reports have been presented. All these findings again indicate the reliability of SMG preservation during neck dissection.

Survival variation is another main concern of SMG-preserving neck dissection. Few authors have aimed to evaluate whether SMG preservation can affect survival in oral SCC. Lanzer et al. (14) noted that in patients with SCC of the FOM or tongue, locoregional recurrence occurred in 28.5% of patients with SMG preservation, and recurrence-free survival was significantly decreased; therefore, the authors concluded that the SMG should not be preserved in patients with SCC of the tongue or FOM. However, in this study, a number of advanced-stage patients had SMG excision, and inappropriate operations might have contributed to the decreased prognosis. Chen et al. (13) depicted that there were similar disease-free survival and overall survival rates between tongue and buccal SCC patients with and without SMG preservation, but because no FOM SCC patients had SMG-preserving neck dissection, the study failed to tell us whether there was a similar trend in FOM SCC patients. Our study was the first to demonstrate that SMG preservation does not have a negative effect on survival in early-stage FOM SCC patients. The finding was significant, and it provides the most important evidence for the feasibility of SMG preservation. However, the effect of different ages and tumor stage distributions between the two groups cannot be ignored, as both variables had a significant impact on prognosis in FOM SCC. Furthermore, owing to the limited sample size, we could not perform a matched-pair analysis.

There were some limitations we must acknowledge: first, this retrospective study had inherent unnoticed selection bias, which might have decreased our statistical power. Second, our number of patients with SMG preservation was small; therefore, more randomized prospective studies with larger sample sizes are needed to clarify the question. Third, we did not compare the quality of life between the two groups, but there have been a few studies describing better saliva production and fewer complaints in patients with SMG preservation (27). However, we must keep in mind that adjuvant radiotherapy is still strongly suggested in patients with SMG preservation if needed, and we could not pursue functional results in patients with compromised disease control (6).

In summary, in early-stage FOM SCC patients, SMG involvement is rare, the most common metastatic site in level 1b metastasis is the superior group, and SMG preservation does not decrease the LRC or DSS rates. Therefore, the findings suggest that there might be high feasibility of SMG-preserving neck dissection in cT1-2N0 FOM SCC.

## Data Availability Statement

The original contributions presented in the study are included in the article/supplementary materials, further inquiries can be directed to the corresponding author/s.

## Ethics Statement

The Zhengzhou University institutional research committee approved our study, and all participants provided written informed consent for medical research prior to initial treatment, and all experiments were performed in accordance with relevant guidelines and regulations.

## Author Contributions

All authors made significant contribution in manuscript design, data collection, data analysis, and manuscript writting and revision.

## Conflict of Interest

The authors declare that the research was conducted in the absence of any commercial or financial relationships that could be construed as a potential conflict of interest.
